# Supplementary figures and images for: RUNX1 facilitates heart failure progression through regulating TGF-β-induced cardiac remodeling
Source: PeerJ. 2023 Oct 31;11:e16202. doi: 10.7717/peerj.16202 (PMC10624168; doi:10.7717/peerj.16202)

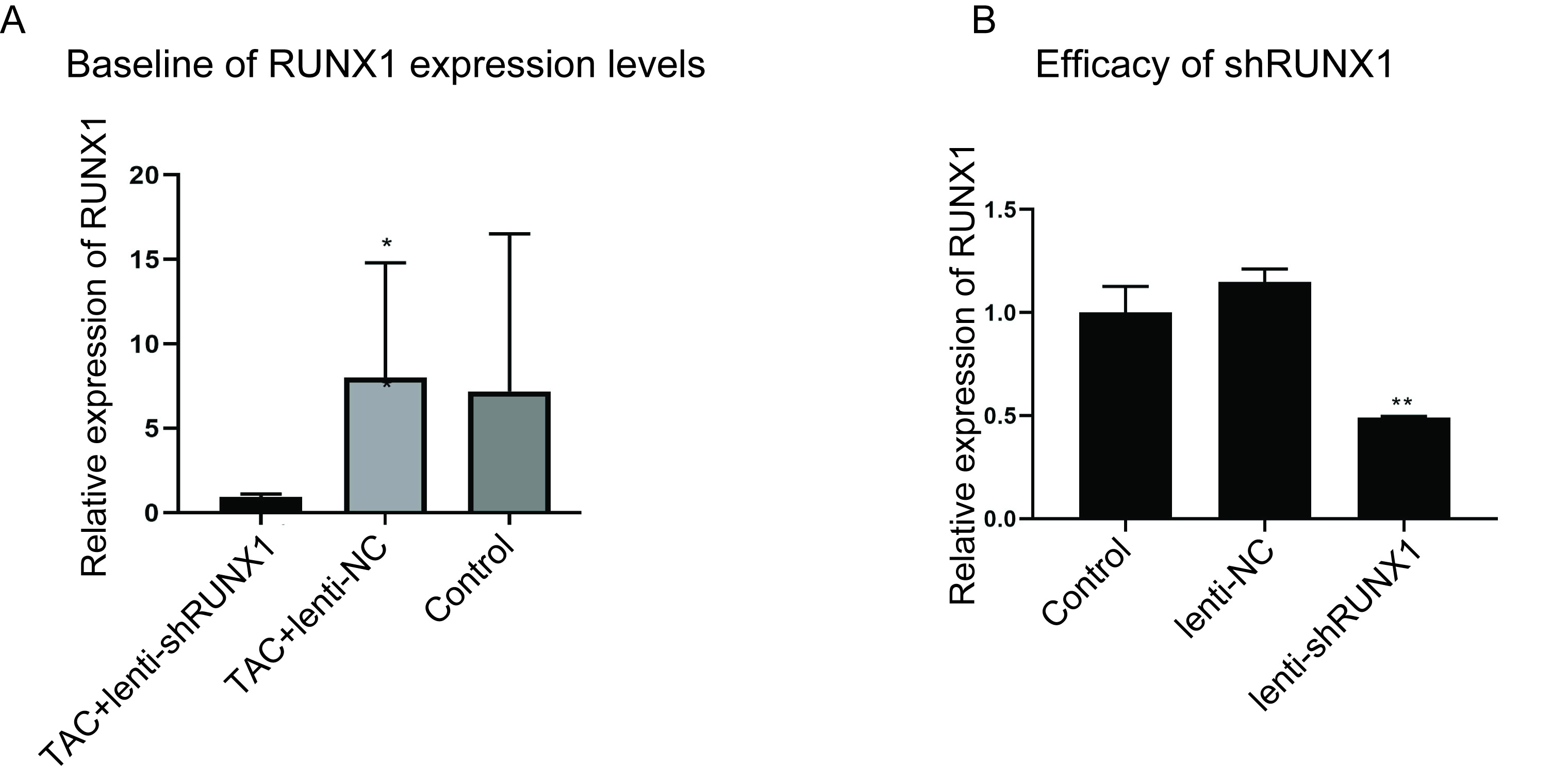

Supplement: Supplemental Information 2 [file peerj-11-16202-s002.jpg]
