# Supplementary material for: RUNX1 facilitates heart failure progression through regulating TGF-β-induced cardiac remodeling
Source: PeerJ. 2023 Oct 31;11:e16202. doi: 10.7717/peerj.16202 (PMC10624168; doi:10.7717/peerj.16202)
Supplement: Supplemental Information 6 [file peerj-11-16202-s006.pdf]

SAFETY DATA SHEET according to GB/T 16483 and GB/T 17519

Version 8.1
Revision Date 22.11.2022
Print Date 21.05.2023
Date of first issue 24.08.2022

SDS No. Millipore - SCC065
Product Number Millipore - SCC065

HL-1 Cardiac Muscle Cell Line

SECTION 1: Identification of the substance/mixture and of the company/undertaking

1.1 Product identifiers

Product name : HL-1 Cardiac Muscle Cell Line

Product Number : SCC065
Catalogue No. : 9QQ0M8
Brand : Millipore

1.2 Details of the supplier of the safety data sheet

Company : Sigma-Aldrich (Shanghai) Trading Co.Ltd.
509 Renqing Road
Zhangjiang High Tech East Park, Pudong
SHANGHAI
201201 SHANGHAI
CHINA

西格玛奥德里奇（上海）贸易有限公司
上海市浦东新区仁庆路 509 号 10 幢
邮政编码：201201

Merck KGaA
64271 Darmstadt
Germany
Phone:+49 6151 72-0

Telephone : +86 21 6141-5566
Fax : +86 21 6141-5567

1.3 Emergency telephone

Emergency Phone # : +86 532 83889090

1.4 Relevant identified uses of the substance or mixture and uses advised against

Identified uses : Biochemical research/analysis

SECTION 2: Hazards identification

Summary of emergency

liquid yellow, brown Combustible liquid. After inhalation: fresh air. In case of skin contact:

The life science business of Merck operates as MilliporeSigma in the US and Canada
MERCK

Take off immediately all contaminated clothing. Rinse skin with water/ shower. After eye contact: rinse out with plenty of water., Remove contact lenses. After swallowing: make victim drink water (two glasses at most). Consult doctor if feeling unwell. Mixture with combustible ingredients. Forms explosive mixtures with air on intense heating. Development of hazardous combustion gases or vapours possible in the event of fire. Violent reactions possible with: strong oxidising agents

2.1 GHS Classification

Flammable liquids (Category 4), H227

For the full text of the H-Statements mentioned in this Section, see Section 16.

2.2 GHS Label elements, including precautionary statements

Pictogram	none
Signal Word	Warning
Hazard statement(s)	
H227	Combustible liquid.
Precautionary statement(s)	
Prevention	
P210	Keep away from heat/ sparks/ open flames/ hot surfaces. No smoking.
P280	Wear protective gloves/ eye protection/ face protection.
Response	
P370 + P378	In case of fire: Use dry sand, dry chemical or alcohol-resistant foam to extinguish.
Storage	
P403 + P235	Store in a well-ventilated place. Keep cool.
Disposal	
P501	Dispose of contents/ container to an approved waste disposal plant.

Reduced Labeling (<= 125 ml)

Pictogram	none
Signal Word	Warning
Hazard statement(s)	
H227	Combustible liquid.
Precautionary statement(s)	none

2.3 Physical and chemical hazards

H227	Combustible liquid.

2.4 Health hazards

Referring to current information, no health hazard.

2.5 Environmental hazards

Referring to current information, no environmental hazard.

2.6 Other hazards - none

SECTION 3: Composition/information on ingredients

Substance / Mixture : Mixture

The life science business of Merck operates as MilliporeSigma in the US and Canada

3.2 Mixtures

Hazardous ingredients

Component		Classification	Concentration
**dimethyl sulphoxide**			
CAS-No.<br>EC-No.	67-68-5<br>200-664-3	Flammable liquids<br>Category 4; H227	>= 1 - < 10<br>%

For the full text of the H-Statements mentioned in this Section, see Section 16.

SECTION 4: First aid measures

4.1 Description of first-aid measures

If inhaled
After inhalation: fresh air.

In case of skin contact
In case of skin contact: Take off immediately all contaminated clothing. Rinse skin with water/ shower.

In case of eye contact
After eye contact: rinse out with plenty of water. Remove contact lenses.

If swallowed
After swallowing: make victim drink water (two glasses at most). Consult doctor if feeling unwell.

4.2 Most important symptoms and effects, both acute and delayed

The most important known symptoms and effects are described in the labelling (see section 2.2) and/or in section 11

4.3 Indication of any immediate medical attention and special treatment needed

No data available

4.4 Notes to physician

No data available

SECTION 5: Firefighting measures

5.1 Extinguishing media

Suitable extinguishing media
Foam Carbon dioxide (CO_2) Dry powder

Unsuitable extinguishing media
For this substance/mixture no limitations of extinguishing agents are given.

5.2 Special hazards arising from the substance or mixture

Carbon oxides
Sulfur oxides
Mixture with combustible ingredients.
Forms explosive mixtures with air on intense heating.
Development of hazardous combustion gases or vapours possible in the event of fire.

5.3 Advice for firefighters

In the event of fire, wear self-contained breathing apparatus.

Remove container from danger zone and cool with water. Suppress (knock down) gases/vapors/mists with a water spray jet. Prevent fire extinguishing water from contaminating surface water or the ground water system.

SECTION 6: Accidental release measures

6.1 Personal precautions, protective equipment and emergency procedures

Advice for non-emergency personnel: Do not breathe vapors, aerosols. Keep away from heat and sources of ignition. Evacuate the danger area, observe emergency procedures, consult an expert.
For personal protection see section 8.

6.2 Environmental precautions

Do not let product enter drains.

6.3 Methods and materials for containment and cleaning up

Cover drains. Collect, bind, and pump off spills. Observe possible material restrictions (see sections 7 and 10). Take up with liquid-absorbent material (e.g. Chemizorb®). Dispose of properly. Clean up affected area.

6.4 Reference to other sections

For disposal see section 13.

SECTION 7: Handling and storage

7.1 Precautions for safe handling

Advice on protection against fire and explosion
Keep away from open flames, hot surfaces and sources of ignition.Take precautionary measures against static discharge.

Hygiene measures
Change contaminated clothing. Wash hands after working with substance.
For precautions see section 2.2.

7.2 Conditions for safe storage, including any incompatibilities

Storage conditions
Tightly closed.

Recommended storage temperature see product label.

Storage class
Storage class (TRGS 510): 10: Combustible liquids

SECTION 8: Exposure controls/personal protection

8.1 Control parameters

Ingredients with workplace control parameters

Component	CAS-No.	Value	Control parameters	Basis
dimethyl sulphoxide	67-68-5	PC-TWA	160 mg/m3	Occupational exposure limits for hazardous agents in the workplace - Chemical hazardous agents.
	Remarks	Skin		

8.2 Exposure controls

Appropriate engineering controls

Change contaminated clothing. Wash hands after working with substance.

Personal protective equipment

Eye/face protection

Use equipment for eye protection tested and approved under appropriate government standards such as NIOSH (US) or EN 166(EU). Safety glasses

Skin protection

This recommendation applies only to the product stated in the safety data sheet, supplied by us and for the designated use. When dissolving in or mixing with other substances and under conditions deviating from those stated in EN374 please contact the supplier of CE-approved gloves (e.g. KCL GmbH, D-36124 Eichenzell, Internet: www.kcl.de).
Full contact
Material: Chloroprene
Minimum layer thickness: 0.65 mm
Break through time: > 480 min
Material tested:KCL 720 Camapren®

This recommendation applies only to the product stated in the safety data sheet, supplied by us and for the designated use. When dissolving in or mixing with other substances and under conditions deviating from those stated in EN374 please contact the supplier of CE-approved gloves (e.g. KCL GmbH, D-36124 Eichenzell, Internet: www.kcl.de).
Splash contact
Material: Latex gloves
Minimum layer thickness: 0.6 mm
Break through time: > 240 min
Material tested:Lapren® (KCL 706 / Aldrich Z677558, Size M)

Respiratory protection

Not required; except in case of aerosol formation.

Control of environmental exposure

Do not let product enter drains.

SECTION 9: Physical and chemical properties

9.1 Information on basic physical and chemical properties

a)	Physical state	liquid
b)	Color	yellow, brown
c)	Odor	No data available
d)	Melting point/freezing point	No data available
e)	Initial boiling point and boiling range	No data available
f)	Flammability (solid, gas)	No data available
g)	Upper/lower flammability or explosive limits	No data available

The life science business of Merck operates as MilliporeSigma in the US and Canada

h)	Flash point	No data available
i)	Autoignition temperature	No data available
j)	Decomposition temperature	No data available
k)	pH	No data available
l)	Viscosity	Viscosity, kinematic: No data available<br>Viscosity, dynamic: No data available
m)	Water solubility	No data available
n)	Partition coefficient: n-octanol/water	No data available
o)	Vapor pressure	No data available
p)	Density	No data available
	Relative density	No data available
q)	Relative vapor density	No data available
r)	Particle characteristics	No data available
s)	Explosive properties	Not classified as explosive.
t)	Oxidizing properties	none

9.2 Other safety information
No data available

SECTION 10: Stability and reactivity

10.1 Chemical stability
The product is chemically stable under standard ambient conditions (room temperature) .

10.2 Possibility of hazardous reactions
Violent reactions possible with:
strong oxidising agents

10.3 Conditions to avoid
Strong heating.

10.4 Incompatible materials
No data available

10.5 Hazardous decomposition products
In the event of fire: see section 5

SECTION 11: Toxicological information

11.1 Information on toxicological effects

Mixture

Acute toxicity
Oral: No data available
Inhalation: No data available
Dermal: No data available

Skin corrosion/irritation
No data available

Serious eye damage/eye irritation
No data available

Respiratory or skin sensitization
No data available

Germ cell mutagenicity
No data available

Carcinogenicity
No data available

Reproductive toxicity
No data available

Specific target organ toxicity - single exposure
No data available

Specific target organ toxicity - repeated exposure
No data available

Aspiration hazard
No data available

11.2 Additional Information

Hazardous properties cannot be excluded but are unlikely when the product is handled appropriately.

Components

dimethyl sulphoxide

Acute toxicity
LD50 Oral - Rat - male and female - 28,300 mg/kg
(OECD Test Guideline 401)
LC0 Inhalation - Rat - male and female - 4 h - > 5.33 mg/l - dust/mist
(OECD Test Guideline 403)
LD50 Dermal - Rat - male and female - 40,000 mg/kg
Remarks: (ECHA)

Skin corrosion/irritation
Skin - Rabbit
Result: slight irritation - 4 h
(OECD Test Guideline 404)

Serious eye damage/eye irritation
Eyes - Rabbit
Result: slight irritation - 24 h

The life science business of Merck operates as MilliporeSigma in the US and Canada

(OECD Test Guideline 405)

Respiratory or skin sensitization
Maximization Test - Guinea pig
Result: negative
(OECD Test Guideline 406)
Local lymph node assay (LLNA) - Mouse
Result: negative
(OECD Test Guideline 429)

Germ cell mutagenicity
Test Type: Ames test
Test system: Salmonella typhimurium
Result: negative
Test Type: sister chromatid exchange assay
Test system: Chinese hamster ovary cells
Result: negative
Test Type: Mutagenicity (mammal cell test): chromosome aberration.
Test system: Chinese hamster ovary cells
Result: negative
Method: OECD Test Guideline 474
Species: Rat - male and female
Result: negative

Carcinogenicity
No data available

Reproductive toxicity
No data available

Specific target organ toxicity - single exposure
No data available

Specific target organ toxicity - repeated exposure

Aspiration hazard
No data available

SECTION 12: Ecological information

12.1 Toxicity

Mixture
No data available

12.2 Persistence and degradability
No data available

12.3 Bioaccumulative potential
No data available

12.4 Mobility in soil
No data available

12.5 Results of PBT and vPvB assessment
PBT/vPvB assessment not available as chemical safety assessment not required/not conducted

12.6 Endocrine disrupting properties
No data available

12.7 Other adverse effects

No data available

Components

dimethyl sulphoxide

Toxicity to fish	static test LC50 - Danio rerio (zebra fish) - > 25,000 mg/l - 96 h<br>(OECD Test Guideline 203)
Toxicity to daphnia and other aquatic invertebrates	static test EC50 - Daphnia magna (Water flea) - 24,600 mg/l - 48 h<br>(OECD Test Guideline 202)
Toxicity to algae	static test ErC50 - Pseudokirchneriella subcapitata (green algae) - 17,000 mg/l - 72 h<br>(OECD Test Guideline 201)
Toxicity to bacteria	EC50 - activated sludge - 10 - 100 mg/l - 30 min<br>(ISO 8192)

SECTION 13: Disposal considerations

13.1 Waste treatment methods

Product

Offer surplus and non-recyclable solutions to a licensed disposal company.

SECTION 14: Transport information

14.1 UN number

ADR/RID: 3373 IMDG: 3373 IATA-DGR: 3373

14.2 UN proper shipping name

ADR/RID: BIOLOGICAL SUBSTANCE, CATEGORY B
IMDG: BIOLOGICAL SUBSTANCE, CATEGORY B
IATA-DGR: Biological substance, Category B

14.3 Transport hazard class(es)

ADR/RID: 3373 IMDG: 3373 IATA-DGR: 3373

14.4 Packaging group

ADR/RID: - IMDG: - IATA-DGR: -

14.5 Environmental hazards

ADR/RID: no IMDG Marine pollutant: no IATA-DGR: no

14.6 Special precautions for user

Based on chemical properties, choose appropriate tools and conditions of transport. Transporting tools shall be equipped with appropriate and sufficient firefighting equipment and emergency leaking installations. If transporting by road, please go along the specified route.

14.7 Incompatible materials

SECTION 15: Regulatory information

15.1 Safety, health and environmental regulations/legislation specific for the substance or mixture

National regulatory information
Law on the Prevention and Control of Occupational Diseases

Measures on the Environmental Administration of New Chemical Substances Registration

Registration/Notification number : B1A222214444

Downstream users need to comply with the conditions of safe use of the chemical, understand the environmental and health hazard and risk management measures identified on the SDS as well as the local/national regulations concerning the chemical.

Other regulations
Please pay attention on the waste treatment should also comply with local regulations requirement.

SECTION 16: Other information

Full text of H-Statements referred to under sections 2 and 3.

H227	Combustible liquid.

Further information
The above information is believed to be correct but does not purport to be all inclusive and shall be used only as a guide. The information in this document is based on the present state of our knowledge and is applicable to the product with regard to appropriate safety precautions. It does not represent any guarantee of the properties of the product. Sigma-Aldrich Corporation and its Affiliates shall not be held liable for any damage resulting from handling or from contact with the above product. See www.sigma-aldrich.com and/or the reverse side of invoice or packing slip for additional terms and conditions of sale.

Copyright 2020 Sigma-Aldrich Co. LLC. License granted to make unlimited paper copies for internal use only.
The branding on the header and/or footer of this document may temporarily not visually match the product purchased as we transition our branding. However, all of the information in the document regarding the product remains unchanged and matches the product ordered. For further information please contact mlsbranding@sial.com.
